# The backbone structure of the thermophilic *Thermoanaerobacter tengcongensis *ribose binding protein is essentially identical to its mesophilic *E. coli *homolog

**DOI:** 10.1186/1472-6807-8-20

**Published:** 2008-03-28

**Authors:** Matthew J Cuneo, Yaji Tian, Malin Allert, Homme W Hellinga

**Affiliations:** 1 The Institute for Biological Structure and Design and the Department of Biochemistry, Duke University Medical Center, Durham, North Carolina, 27710, USA

## Abstract

**Background:**

Comparison of experimentally determined mesophilic and thermophilic homologous protein structures is an important tool for understanding the mechanisms that contribute to thermal stability. Of particular interest are pairs of homologous structures that are structurally very similar, but differ significantly in thermal stability.

**Results:**

We report the X-ray crystal structure of a *Thermoanaerobacter tengcongensis *ribose binding protein (tteRBP) determined to 1.9 Å resolution. We find that tteRBP is significantly more stable (^*app*^*T*_*m *_value ~102°C) than the mesophilic *Escherichia coli *ribose binding protein (ecRBP) (^*app*^*T*_*m *_value ~56°C). The tteRBP has essentially the identical backbone conformation (0.41 Å RMSD of 235/271 C_α _positions and 0.65 Å RMSD of 270/271 C_α _positions) as ecRBP. Classification of the amino acid substitutions as a function of structure therefore allows the identification of amino acids which potentially contribute to the observed thermal stability of tteRBP in the absence of large structural heterogeneities.

**Conclusion:**

The near identity of backbone structures of this pair of proteins entails that the significant differences in their thermal stabilities are encoded exclusively by the identity of the amino acid side-chains. Furthermore, the degree of sequence divergence is strongly correlated with structure; with a high degree of conservation in the core progressing to increased diversity in the boundary and surface regions. Different factors that may possibly contribute to thermal stability appear to be differentially encoded in each of these regions of the protein. The tteRBP/ecRBP pair therefore offers an opportunity to dissect contributions to thermal stability by side-chains alone in the absence of large structural differences.

## Background

The mechanisms that contribute to protein thermal stability are varied, subtle, and complex [1-5]. Various contributing factors to thermal stability have been proposed by comparative analysis of thermophilic and mesophilic proteins [4,6]. Proposed mechanisms can be categorized [5] generally as contributions by the main-chain structure (new folds [7], loop shortening [8]), or by side-chain interactions (increased packing in core [9] or surface [10], alteration of amino acid composition [11-13]), post-translational modifications [14] or co-factor binding [4,15]). Usually increased stability arises from a combination of sequence- and structure-based adaptations resulting in a collection of improvements in the thermophilic protein compared to its mesophilic counterpart [4,6,16,17]. Consequently, the determination of rules for thermal adaptations are difficult to dissect [6]. Of particular interest, therefore, are pairs of naturally evolved proteins that are structurally very similar but differ substantially in thermal stability. Such pairs allow for the dissection of contributions by amino acid diversity to thermal stability in the absence of structural heterogeneity [17-20]. The structure of the *Thermoanaerobacter tengcongensis *ribose-binding (tteRBP) presented here reveals that this protein and its counterpart in the mesophilic *Escherichia coli *(ecRBP) form such a pair.

The ribose-binding proteins are members of the periplasmic binding protein (PBP) superfamily whose members play roles in prokaryotic ABC transport [21], chemotaxis [22,23], and intercellular communication [24] systems. The PBP fold consists of two domains each of which adopts a three-layered α/β/α sandwich motif [25]. The two domains are linked by two or three β-strands that form a flexible hinge which permits the domains of the protein to bend towards each other in response to ligand binding at the interface between the two domains [26-28].

Here we report the high-resolution X-ray crystallographic structure of a ribose binding protein (tteRBP) from the hyperthermophilic bacterium *T. tengcongensis *(optimal growth temp ~80°C) [29]. We find that tteRBP has high sequence and structural similarity to the mesophilic *E. coli *RBP (ecRBP), although they differ markedly in their thermal stability. The near identity of backbone structure offers an opportunity to address local encoding of thermal stability by amino acid substitutions.

## Results and Discussion

### Thermal Stability and Ligand Binding

**Figure 1:**
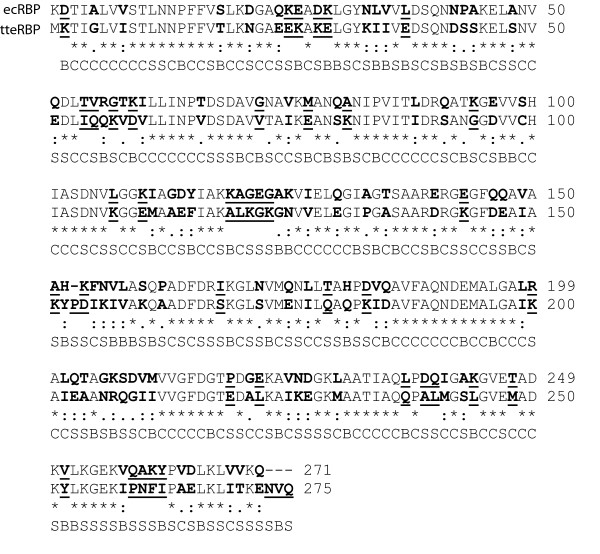
**Amino acid sequence comparison of tteRBP and ecRBP**. Clustal-W amino acid sequence alignment of tteRBP and ecRBP. Amino acids which are not conserved are in bold type and underlined, amino acids that are conserved but not identical are in bold type (charge inversions are scored as non-conservative here). Core, boundary or surface classification of amino acids is shown below the aligned residues.

ORF *tte0206 *in the *T. tengcongensis *genome sequence [29] was postulated to be a ribose-binding protein homolog (tteRBP) based on its sequence similarity to the known *E. coli *RBP (57% identity, 76% similarity) (Figure 1) and its position within a putative operon containing ORFs homologous to ABC transporters characteristic of solute transport. The DNA for ORF *tte0206*, lacking a putative periplasmic signal sequence [30] (residues 1–39), was amplified from *T. tengcongensis *genomic DNA by the polymerase chain reaction. The resulting DNA fragment was cloned into a pET21a vector in-frame with a C-terminal hexa-histidine tag preceded by a glycine-serine linker. The nucleotide sequence was confirmed by DNA sequencing of the resulting vector. Over-expression of *tte0206 *produced ~30 mg of pure protein per liter of medium, which was purified by immobilized metal affinity chromatography followed by gel filtration chromatography. tteRBP eluted from the gel filtration column as a broad peak immediately following the void volume of the column (data not shown). For subsequent crystallization and characterization of tteRBP fractions of the broad peak from the gel filtration column, that were consistent with monomeric tteRBP, (fractions with a calculated hydrodynamic radius of 30 kDa ± 15 kDa) were pooled and concentrated to ~15 mg/mL (see Materials and Methods).

**Figure 2:**
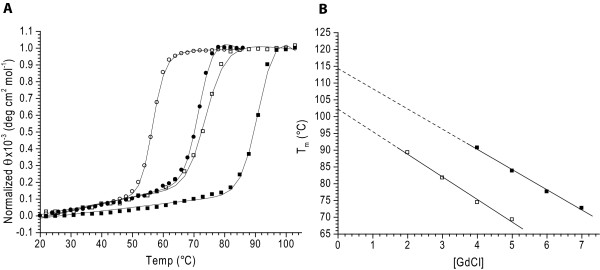
**Thermal denaturation of tteRBP and ecRBP determined by circular dichroism**. (A) Thermal denaturation of tteRBP in 4 M GdCl in the absence (open squares) or presence of 1 mM ribose (black squares). Thermal denaturation of ecRBP in the absence (open circle) or presence of 1 mM ribose (black circles). Solid lines in (A) are fit to a two-state model [31, 32] which takes into account the native and denatured baseline slopes. (B) Extrapolated ^*app*^*T*_*m *_of tteRBP in the absence (open squares) or presence of 1 mM ribose (black squares) obtained from a series of thermal melting curves at different GdCl concentrations. Solid lines represent linear fits to the observations.

The thermal stability of tteRBP was determined by thermal denaturation using circular dichroism (CD). In the absence of denaturant no significant temperature-dependent change in the CD signal was observed up to 100°C; consequently, heat denaturations were carried out in the presence of varying concentrations of guanidine hydrochloride (GdCl) (Figure 2). Melting curves were found to fit a two-state model [31,32]. The apparent thermal transition midpoint (^*app*^*T*_*m*_) of 102°C in the absence of GdCl was determined by linear extrapolation of a series of melting point determinations carried out at different GdCl concentrations [33] (Figure 2). tteRBP is therefore significantly more stable than the mesophilic ecRBP (^*app*^*T*_*m*_value is 56°C) (Figure 2).

Binding of ribose to tteRBP was confirmed by observing ligand-mediated changes in the ^*app*^*T*_*m *_value in the presence of 4.0 M GdCl. Under these conditions in the absence of ribose, the ^*app*^*T*_*m *_value is 74°C; and 92°C in the presence of 1 mM ribose (Figure 2). The ^*app*^*T*_*m *_value of the ribose complex in the absence of GdCl is 114°C; the ^*app*^*T*_*m *_value for ecRBP under equivalent conditions is 72°C (Figure 2).

### Overall Structure of tteRBP

**Figure 3:**
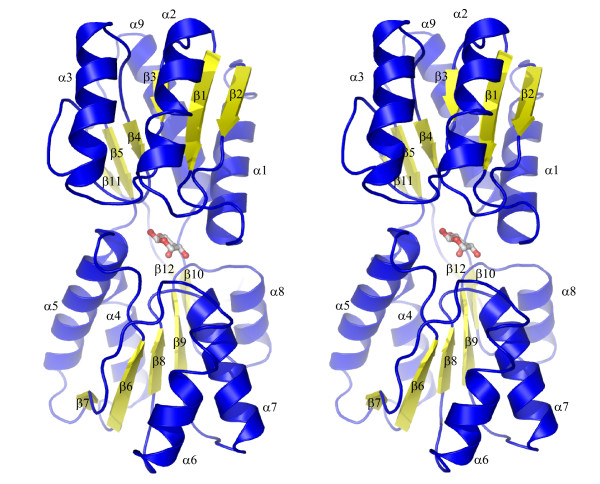
**Stereo diagram of the tteRBP structure**. Ribose is shown in ball-and-stick representation. The ordering of β-strands (yellow) and α-helices (blue) is indicated.

The tteRBP crystal structure was solved to 1.9 Å resolution by molecular replacement [34] using the ribose-bound form of ecRBP as the search model [23]. The tteRBP structure adopts the overall fold and topology that is characteristic of periplasmic ribose-binding proteins (Figure 3). The asymmetric unit contains 346 water molecules and two tteRBP molecules (residues 40–313) in essentially identical conformations (0.12 Å RMSD of backbone atoms) complexed with ribose. Data collection, stereochemistry, and refinement statistics are summarized in Table 1.

**Table 1:** Data collection and refinement statistics

	**tteRBP**
**Data Collection**	
Detector Type	Mar225
Wavelength (Å)	1.0
Resolution (Å)	15.0–1.9
Measured reflections	123829
Unique reflections	16732
Mean I/σ(I)^a^	11.6 (4.1)
Completeness (%)^a^	96.0 (95.1)
R_sym _(%)^a,b^	8.0 (32.4)
Redundancy^a^	3.3 (3.1)
**Refinement**	
Resolution (Å)	15.0–1.9
Num. of Reflections (working set/test set)	35702/1890
R_cryst_^c^	19.9 (26.0)
R_free_^d^	23.4 (30.6)
Number of atoms	
Protein	4265
Water	346
Ligand	20
**r.m.s.d.**	
Bond lengths (Å)	0.011
Bond angles (°)	1.221
**Average B-factor **(**Å**^**2**^)	
Main Chain	17.8
Side Chain	22.7
Solvent	28.7
Ligand	10.1
**Protein Geometry**	
Ramachandran outliers (%)	0.55
Ramachandran favored (%)	98.7
Rotamer outliers (%)	0.47

^a^Number in parentheses represent values in the highest resolution shell.

^b^R_sym _= ∑|(I-<I>)|/(∑I), where <I> is the average intensity of multiple measurements.

^c^R_cryst _= ∑|F_obs_-F_calc_|/(∑|F_obs_|)

^d^R_free _is the R-factor based on 5% of the data excluded from refinement.

### Structural Diversity of tteRBP and ecRBP

**Figure 4:**
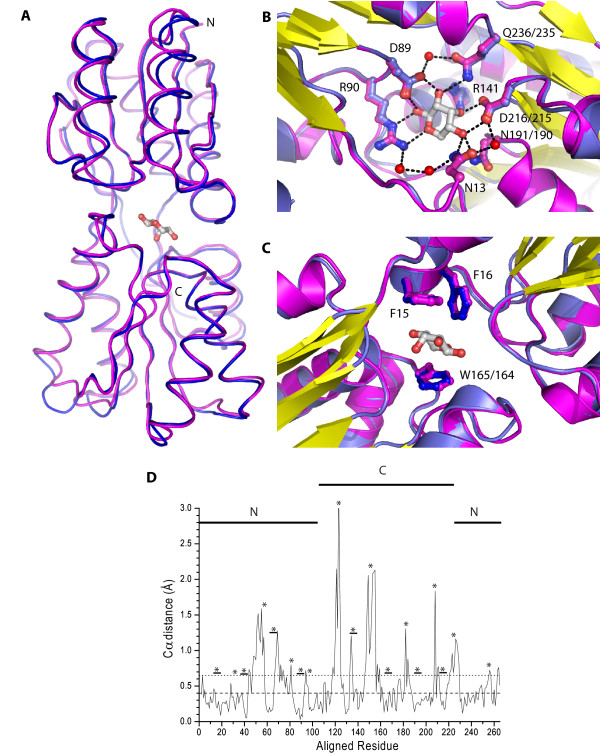
**Similarity between ecRBP and tteRBP**. (A) Backbone atom alignment of tteRBP (blue) and ecRBP (magenta). Loops which have high RMSD are indicated (1/residues 55–61, 2/residues 117–126, 3/residues 149–156). (B) Close-up view of the polar binding pocket residues in tteRBP (blue) and ecRBP (magenta). Ribose is shown in gray. Critical residues involved in ribose binding are indicated (where the tteRBP and ecRBP numbering are different, the former is given first). (C) Close-up view of the non-polar binding pocket amino acids of tteRBP (blue) and ecRBP (magenta). (D) Structural differences in the Cα positions of the aligned models of ecRBP and tteRBP generated by LSQMAN [60]. Dashed and dotted lines indicate the RMSD of 235/271 and 270/271 of the Cα atoms respectively of the aligned structures. The N- and C- terminal residues are indicated with a solid line. Loops and turns are indicated (asterisk), or loops (underlined asterisk) in the binding pocket region.

Analysis of main-chain and side-chain geometry of the aligned structures indicates there are few differences in the main-chain geometries of ecRBP and tteRBP (0.4 Å RMSD of 235/271 C_α _positions and 0.65 Å RMSD of 270/271 C_α _positions and distance between aligned C_α _positions range from 0.03–3.1 Å over 270 Cα positions). The loops and turns in the binding pocket retain near-identical conformations. Modest backbone conformational heterogeneity is observed in loops and turns that connect alternating β-strands and α-helices in tteRBP and ecRBP (RMSD of C_α _positions for residues 55–61 is 0.9 Å, 117–126 is 1.6 Å and 149–156 is 3.02 Å) (Figure 4). Proline 153 in tteRBP corresponds to a single-residue insertion relative to ecRBP; small structural perturbations associated with this insertion are contained within five amino acids preceding and following this residue (3.1 Å RMSD of C_α _positions). tteRBP also contains an additional three amino acids at the C-terminus that are not present in ecRBP.

The amino acid side-chain conformations are also remarkably well conserved (Table 2). Only 24 residues show a significant change in the χ_1 _torsion, resulting in the adoption of a different side-chain rotamer. Of these, 19 correspond to substitutions, including non-conservative changes; there is therefore a significant bias for non-conservative mutations in the population of residues that exhibit rotameric changes. 17 of the rotamer changes occur in the surface, three in the boundary, and four in the core (Table 2). The non-conservative changes occur mostly on the surface (seven residues). Two of the surface rotamer changes (Q24/E24, D182/D183) involve charged amino acids which results in the formation of two salt bridges, two involve the loss of a salt bridge (K110/E110, K243/L244), one involves the loss of a hydrogen-bond (T178/Q179) and three positions are involved gain of five additional hydrogen-bonds (D52, Q80/S80, R139) relative to ecRBP. The four core changes are conservative substitutions and involve β-branched amino acids, altering packing of the core (V8/I8, I60/V60, T66/V66, V183/I184), and in one case (T66/V66) increasing the hydrophobicity and removing an unsatisfied core hydrogen-bond.

**Table 2:** Changes in the χ_1 _values of ecRBP and tteRBP. Non-conservative substitutions (as defined in [36]; charge inversions are scored as non-conservative here) are underlined.

ecRBP Residue	tteRBP Residue	ΔX_1_	Classification (C/B/S)
VAL 8	ILE 8	137	C
GLN 24	GLU 24	-97	S
LYS 25	GLU 25	-100	S
ASN 33	LYS 33	-106	S
ASP 52	ASP 52	-283	S
ILE 60	VAL 60	-115	C
THR 66	VAL 66	-114	C
GLN 80	SER 80	102	S
GLN 91	SER 91	-193	S
VAL 98	VAL 98	-120	B
LYS 110	GLU 110	244	B
LYS 118	LYS 118	236	S
ARG 139	ARG 139	-99	S
GLN 147	GLU 147	-240	S
ASN 155	LYS 156	-135	S
THR 178	GLN 179	-93	S
ASP 182	LYS 183	-125	S
VAL 183	ILE 184	125	C
ARG 199	LYS 200	254	S
GLN 202	GLU 203	246	S
SER 207	GLN 208	110	S
GLN 239	LEU 240	-246	S
LYS 243	LEU 244	-92	B
GLU 246	GLU 247	-103	S

Polar amino acids, non-polar amino acids, waters, and the hydrogen-bonding interactions are identical in both tteRBP and ecRBP sugar-binding pockets (Figure 4). The total number of hydrogen-bonding interactions [35] is also well conserved among tteRBP and ecRBP (Table 3). Overall, tteRBP has a total of 264 hydrogen-bonds, ecRBP has 257. The hydrogen-bonding pattern outside of the binding pocket varies slightly among tteRBP and ecRBP. tteRBP has an additional three side-chain/main-chain and nine main-chain/main-chain hydrogen-bonds, but has lost five side-chain/side-chain hydrogen-bonds relative to ecRBP (Table 3). Five of the additional seven hydrogen-bonds observed in tteRBP (two main-chain/side-chain, three main-chain/main-chain) are accounted for by the four-residue insertion in tteRBP. There is therefore a net gain of two hydrogen-bonds in tteRBP, which arise from the slight differences in the hydrogen bonding pattern of the side-chain/side-chain and main-chain/main-chain residues. It is also observed that tteRBP has lost two salt bridges relative to ecRBP.

**Table 3:** Hydrogen bonding interactions in tteRBP and ecRBP

**Class**	**tteRBP**	**ecRBP**
Side chain/Side chain	28	33
Salt Bridges	8	10
Side chain/Main chain	44	41
Main chain/Main chain	192	183

### Amino Acid Diversity Among tteRBP and ecRBP

**Figure 5:**
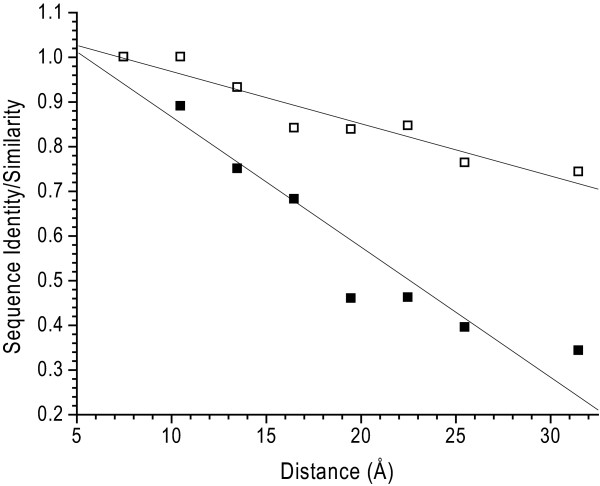
**Structure based sequence comparison of ecRBP and tteRBP**. Comparison of sequence identities (black squares) and similarities (open squares) of tteRBP and ecRBP by scoring the number of identical residues in 3 Å concentric shells centered on the mid-point of the bound-ribose. The residues in the last two bins were combined due to the few members in the largest bin. In the primary complementary surface (first shell, 9 Å), the two sequences are identical; at the furthest distance the two sequences are 34% identical and 74% similar. Solid lines are a linear fit of the data to the observations.

The two RBPs share 57% amino acid identity and 76% similarity (as defined in [36]; in our study charge inversions are scored as non-conservative) (Figure 1). The structures can be divided into core (C), boundary (B), and surface (S) regions using an objective, structure-based classification scheme [37] (Figure 1 and Table 4). The conditional probability of a substitution occurring in a particular region (R) of the protein, *p*(M|R), is strongly biased (*g*(M|R) = *p*(M|R)|*p*(R)), with *g*(M|C) = 0.53, *g*(M|B) = 2.7 and *g*(M|S) = 1.6 for all interactions and 0.21, 1.36, and 1.74 respectively for non-conservative mutations (*g*<1, anticorrelated; *g *= 1, uncorrelated; *g*>1, positively correlated). The pattern of sequence divergence is also correlated with the distance from the ribose-binding site, as measured by the sequence identity and similarity, in a series of concentric shells centered on the bound ribose (Figure 5). Not surprisingly, the residues in the shell forming the ribose contacts are identical. With increasing distance, there is an approximately monotonic decrease in sequence identity and similarity, with the farthest shell having 73% and 34% similarity and identity respectively (Figure 5).

**Table 4:** Divergence patterns in tteRBP and ecRBP. Classification of amino acids into core, boundary, or surface [37] allows identification of the regions which are conserved among tteRBP and ecRBP.

		**Mutations**	
		
**Region**	**Residues**	**All**	Non-conservative*
**Core**	118	28	5
**Boundary**	58	33	16
**Surface**	99	58	35

**Sum**	275	119	56

*As defined in [36]; charge inversions are scored as non-conservative here.

Analysis of the amino acid diversity among the core, boundary and surface of ecRBP and tteRBP allows identification of possible determinants of thermal stability in tteRBP (Table 5). A bias is observed for a gain of polar and charged amino acids on the surface of tteRBP (net of twelve charged and three polar substitutions), while the opposite is observed for the tteRBP core, where there is a bias for the loss of polar amino acids (seven net substitutions). There are also a significant number of substitutions of non-β-branched amino acids for β-branched amino acids in the core and boundary of tteRBP (five net substitutions) and a loss of β-branched amino acids in the surface of tteRBP (four net substitutions). Interestingly a large number of β-branched amino acids are conserved in the core and boundary of tteRBP and ecRBP, there is however a bias for the substitution of valine for isoleucine in the thermophile (eight net substitutions).

**Table 5:** Amino acid sequence divergence as a function of core, boundary or surface in tteRBP and ecRBP. Differences are classified as substitutions which are found in tteRBP relative to ecRBP.

**Classification**	**Lose Charge**	**Lose Polar**	**Gain Charge**	**Gain Polar**	**Non-Branched to Branched**	**Branched to Non-Branched**	**V to I**	**I to V**
Core	1	7	1	0	5	4	7	2
Boundary	4	3	4	5	6	2	3	0
Surface	6	9	18	12	0	4	0	0

## Conclusion

We have cloned, expressed, purified, and characterized the structure and stability of the ribose binding protein from the extremophilic bacterium *T. tengcongensis*. tteRBP is considerably more stable than ecRBP (46°C difference in ^*app*^*T*_*m *_values of the apo proteins). The amino acid backbone structure of these two proteins are essentially identical (0.41 Å RMSD of 235/271 C_α _positions and 0.65 Å RMSD of 270/271 C_α _positions), suggesting that all the interactions contributing to differences in thermal stability are encoded entirely in the identity, location, and conformation of the amino acid side-chains.

Comparison of mesophilic and thermophilic protein structures has identified many structural adaptations which are postulated to confer thermal stability [2,6,11,16-18,38]. Numerous side-chain dependent contributions to thermal stability have been proposed, based on amino acid composition of thermophilic proteins and comparison of mesophilic and thermophilic protein sequences and structures, including; increased number of salt-bridges [8], differences in polar/apolar exposed and buried surface areas [8,12,39], introduction of prolines [40], introduction of disulfide bridges [41,42], aromatic interactions [8], helix dipole stabilization [43], post-translational modification [14], alteration of amino acid packing [9,10,44] and secondary structure propensity of amino acids [8,45].

The high structural similarity of the tteRBP/ecRBP pair allows for the dissection of amino acid diversity contributions to thermal stability in the absence of structural heterogeneity. The comparative analysis presented here shows that the substitutions responsible for conferring thermal stability on tteRBP are encoded in side-chain identity and location (core, boundary or surface) which serves to alter surface polarity/charge, removal of unsatisfied core hydrogen bonds and increase in core/boundary side-chain hydrophobicity. In the core of tteRBP there is a bias for the loss of polar amino acids and for the introduction of valine to isoleucine mutations which possibly lower the entropic contribution to the free energy of folding and limits burying core amino acids whose hydrogen bonding potential may remain unsatisfied [38,46]. The large number of valine to isoleucine substitutions in the tteRBP core and boundary leads to an increase in side-chain hydrophobicity and increased packing [44,47]. It is additionally observed in the boundary the substitution of non-β-branched amino acids for β-branched residues which has also been postulated to be important in increasing the packing [48]. Additionally, in a trend that is also observed in other thermophilic proteins, the surface of tteRBP is generally more polar and charged with the introduction of an additional three polar residues and eleven charged residues.

The acquisition of thermal stability in tteRBP arises from contributions by side-chain mediated effects alone. This pair of proteins therefore provides a good test case to examine such contributions experimentally and address some long-standing questions in the acquisition of protein stability [1,5,49]: where in sequence and structure is stability encoded; how many mutations are needed; are mutations punctuated (single mutants cause large changes) or gradual, independent or correlated? Recent advances in protein fabrication automation [50] will assist in addressing these questions by enabling rapid construction of the many sequence variants needed.

## Methods

### Cloning Over-expression and Purification

The *tte0206 *gene was amplified from *T. tengcongensis *genomic DNA by the sticky-end PCR method using the following primers: PO_4_-TATGA AAACTATAGG ATTAGTGATATCTACTCTTAACAATCC, and TATGAAAACTATAGG ATTAGTGATATCTACTCTTAACAATCC for the 5' end of the gene; PO_4_- AATTCTAATGGTGATGGTGATGGTGTGATCCCTGTACATTTTCTTTTGTTATGAGTTTAAGTTCTGC, and CTAATGGTGATGGTGATGGTGTGATCCCTGTACATTTTCTTTTGTTATGAGTTTAAGTTCTGC for the 3' end of the gene [51]. The resulting fragment was cloned into the *Nde*I/*Eco*RI sites of a pET21a (Novagen) plasmid for over-expression in *E. coli*. This ORF lacks the putative periplasmic signal sequence [30]. The coding sequence starting at lysine 40 was cloned in-frame with an ATG start codon. A hexahistidine affinity tag and a glycine-serine linker was fused in-frame at the carboxy terminus to facilitate purification by immobilized metal affinity chromatography (IMAC). Protein concentration was determined spectrophotometrically (ε_280 _= 3800 M^-1^cm^-1^) [52]. The resulting gene product was expressed and purified by IMAC as described [33]. Pooled IMAC fractions were concentrated to 12 mL and were loaded onto a Superdex 26/60 S75 (Amersham) gel filtration column that was previously calibrated with blue dextran, bovine serum albumin, chicken serum albumin, chymotrypsin and lysozyme. tteRBP eluted from the column beginning at the void volume and ending at a calculated hydrodynamic radius corresponding to ~20 KDa. For crystallization and characterization, 10 mL fractions corresponding to a calculated hydrodynamic radius corresponding to an apparent molecular weight of 30 KDa ± 15 kDa, were collected and concentrated to 0.5 mM and dialyzed in 10 mM Tris pH7.8, 20 mM NaCl. An average of 30 mg of pure protein produced per liter of medium.

### Circular Dichroism

Circular dichroism (CD) measurements were determined on an Aviv Model 202 circular dichroism spectrophotometer. Thermal denaturations were determined by measuring the CD signal at 222 nm (1 cm path length) as a function of temperature, using 1 μM protein (10 mM Tris-HCl pH7.8, 150 mM NaCl), GdCl at various concentrations, in the presence or absence of 1 mM ribose. Protein samples were incubated for 15 minutes prior to collecting data. Each measurement includes a 3-second averaging time for data collection and a 60 second equilibration period at each temperature. Data was fit to a two-state model which accounts for the native and denatured baseline slopes, to determine the apparent *T*_*m *_values [31,32]. It is not known whether equilibrium was achieved under these conditions; denaturation midpoint temperatures are therefore reported as apparent values (^*app*^*T*_*m*_). The ^*app*^*T*_*m *_values in the absence of denaturant were determined by linear extrapolation [33].

### Crystallization and Data Collection

Ribose was added to tteRBP in 3-fold stoichiometric excess prior to crystallization. tteRBP crystals were grown by micro-batch under paraffin oil in drops that contained 2 μl of the protein solution (0.5 mM) mixed with 2 μl of 0.1 M sodium citrate pH 4.0, 50% (w/v) PEG 1000 and 0.1 M potassium phosphate monobasic. The tteRBP crystals diffract to 1.9 Å resolution, belong to the C2 space group (*a *= 123.18 Å, *b *= 35.8 Å, *c *= 118.03 Å, β = 107.02) and typically grew within three weeks at 17°C (Table 1). No stabilizing cryoprotectant was used and crystals were frozen directly in precipitant solution, mounted in a nylon loop and flash frozen in liquid nitrogen. All data were collected at 100 K at the SER-CAT 22 BM beam line at the Advanced Photon Source. The diffraction data were scaled and indexed using SCALA and XDS [53,54].

### Structure Determination Methods, Model Building and Refinement

The tteRBP structure was determined by molecular replacement using the ribose-bound form of the ribose binding protein from *E. coli *[23] as the search model [34]. Rotation, translation, and fitting functions revealed a clear solution yielding higher correlation coefficients and a lower *R *factor than all the others. Manual model building was carried out in the programs O and COOT and refined using REFMAC5 [55-57]. The final model for the tteRBP complex includes two intact tteRBP monomers (residues 2–275), two ribose molecules, and 346 water molecules. The model exhibits good stereochemistry as determined by PROCHECK and MolProbity; final refinement statistics are listed in Table 1[58,59]. PDB coordinates and structure factors have been deposited in the RCSB Protein Data Bank under the accession code 2IOY.

## Authors' contributions

YT constructed the original clone and carried out circular dichroism experiments on purified tteRBP. MJC purified, crystallized and solved the structure of tteRBP, and carried out circular dichroism experiments on ecRBP. MJC, MA and HWH undertook sequence and structural analysis of the tteRBP and ecRBP structures. MJC and HWH wrote the manuscript. All authors have read and approved the final manuscript.
